# Resection of chronic expanding hematoma within the liver cyst difficult to differentiate from liver cystadenocarcinoma: A case report

**DOI:** 10.1002/ccr3.8294

**Published:** 2023-12-05

**Authors:** Kodai Abe, Shunsuke Tomita, Shohei Suzuki, Yu Kigasawa, Hironobu Kashiwagi, Michihito Nishioka, Yasuo Kabeshima

**Affiliations:** ^1^ Department of Surgery Isehara Kyodo Hospital Isehara Japan

**Keywords:** chronic expanding hematoma, cystadenocarcinoma, liver cyst

## Abstract

Liver cysts are common benign lesions with rare malignancy potential. Distinguishing between benign and malignant tumors within liver cysts is challenging. We present the case of a patient with a chronically expanding hematoma within a liver cyst that was resected under suspicion of liver cystadenocarcinoma. A 73‐year‐old female patient presented with elevated hepatobiliary enzyme levels, no viral hepatitis, elevated tumor marker levels, and preserved liver capacity (Child–Pugh grade A). Abdominal ultrasonography revealed a large cyst (>10 cm) occupying the right lobe and a 25‐mm mass lesion with mixed echogenicity inside the cyst. Contrast‐enhanced computed tomography showed atrophy of the parenchyma of the right lobe and dilation of the right intrahepatic bile duct due to the large cyst. Moreover, in the arterial phase, a point‐like high‐density area was observed inside the nodule, which increased from 25 to 35 mm over 3 months. Diffusion‐weighted magnetic resonance imaging revealed a high‐intensity signal within the nodule; however, positron emission tomography did not show an increased accumulation of fluorodeoxyglucose in the same area. Considering the risk of peritoneal dissemination if the cyst was punctured and found to be malignant, we performed a right hepatectomy. Pathological findings revealed a brownish fluid‐filled cyst containing a dark reddish nodule diffusely filled with hematoma, confirming the absence of a malignancy. To date, the patient has not experienced recurrence. We encountered a case of a chronic, expanding hematoma originating from a liver cyst that was difficult to distinguish preoperatively from a liver cystadenocarcinoma.

## Graphical abstract

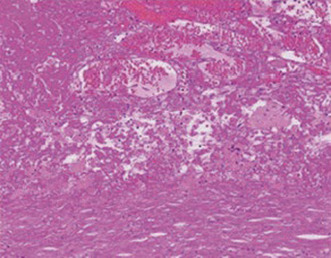

## 1 INTRODUCTION

Liver cysts are common worldwide, with the majority of individuals remaining asymptomatic. These benign lesions typically exhibit minimal or no changes in size or shape over time. However, some liver cysts can cause hemorrhage, which may be triggered by external factors like bruises and falls. Hematomas resulting from liver cysts are rare, comprising approximately 10% of all cases.^1^ Furthermore, liver cyst hematomas exhibit various imaging features, making it challenging to distinguish between benign and malignant lesions.^2^ Herein, we report a case of a patient with chronic expanding hematoma (CEH) originating within a liver cyst that was difficult to differentiate from malignant cystadenocarcinoma before resection.^3^

## 2 CASE REPORT

A 73‐year‐old woman was referred to our hospital following a physical examination that indicated elevated hepatobiliary enzyme levels in her blood sample. Additionally, an abdominal ultrasound scan revealed a cystic lesion occupying the right lobe of the liver. She was asymptomatic and had no history of smoking or alcohol consumption. Furthermore, she had a history of acute appendicitis and left‐sided breast cancer; however, she had no history of falls or trauma. Her older sister died of cholangiocellular carcinoma, and she was undergoing treatment for bilateral metachronous breast cancer. Blood test results showed a slight elevation in biliary enzyme levels (alkaline phosphatase and gamma‐glutamyl transpeptidase); however, hepatic enzyme levels were within the normal range. The patient was assigned a Child–Pugh score of A (5 points), and the indocyanine green test score at 15 min was 5%, indicating a good reserve capacity of the liver. Furthermore, hepatitis virus markers were negative, and tumor markers (alpha‐fetoprotein, protein induced by vitamin K absence‐II, carcinoembryonic antigen (CEA), carbohydrate antigen 19‐9 [CA19‐9]) were within the normal range.

**FIGURE 1:**
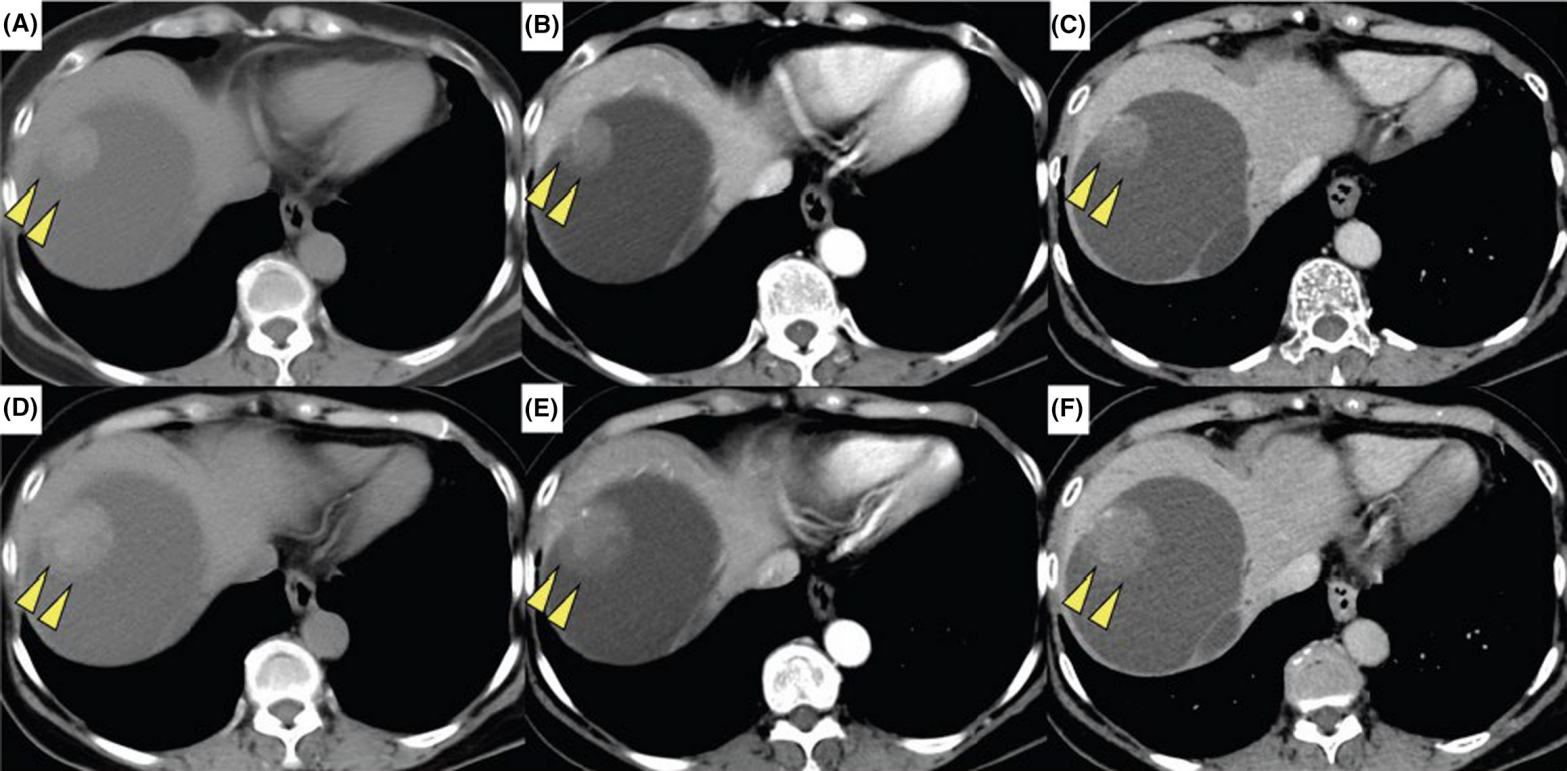
Preoperative computed tomography findings. Computed tomography conducted 4 months preoperatively (A–C) and computed tomography conducted 1 month preoperatively (D–F) reveal a 1.5‐fold increase in tumor size during the 3‐month period. The nodule within the liver cyst has a slightly higher density than the liver parenchyma (A, D), and a punctate high‐density area inside the nodule in the arterial phase (B, E) and delayed phase (C, F).

**FIGURE 2:**
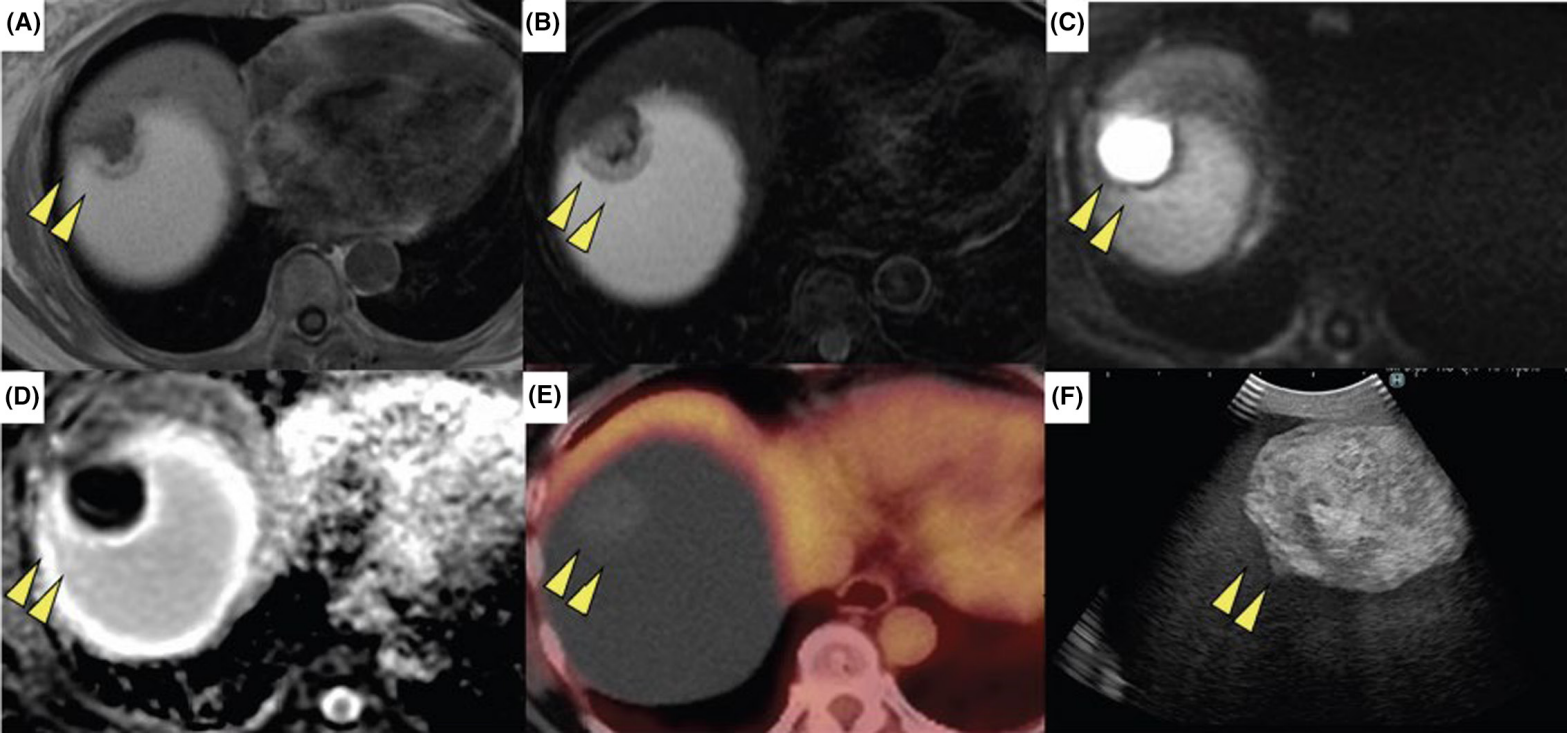
Preoperative magnetic resonance imaging (MRI) and positron emission topography (PET). (A) T1 weighted MRI scan shows a low signal intensity in the nodule within the liver cyst. (B) T2 weighted MRI scan shows an intermediate signal intensity in the nodule. (C) Diffusion‐weighted MRI scan reveals a high signal intensity in the tumor. (D) Apparent diffusion coefficient map MRI showing low signal intensity in the tumor. (E) PET shows no obvious hyperaccumulation of fluorodeoxyglucose in the nodule. (F) Intraoperative ultrasonography reveals uniform high echoic lesion with clear boundary.

Contrast‐enhanced computed tomography showed that the cyst had almost completely replaced the right lobe of the liver; however, there was no obvious evidence of distant metastasis (Figure 1A–F). The size of the liver cyst remained stable over the course of 3 months; however, the internal nodule exhibited a 1.5‐fold increase in size (25–35 mm) (Figure 1A–F). Furthermore, the nodule within the liver cyst had a slightly higher density than that of the liver parenchyma (Figure 1A,D). Additionally, the nodule exhibited a punctate high‐density area within it in the arterial (Figure 1B,E) and delayed phases (Figure 1C,F). Magnetic resonance imaging of the interior of the nodule revealed a mixture of liquid and parenchymal components. The nodule within the liver cyst showed low intensity on T1‐weighted imaging (Figure 2A), intermediate intensity on T2‐weighted imaging (Figure 2B), high intensity on diffusion‐weighted imaging (Figure 2C), and low intensity on apparent diffusion coefficient map imaging (Figure 2D). Fluorodeoxyglucose positron emission tomography revealed no obvious hyperaccumulation in the nodule (Figure 2E).

Despite considering the possibility of a hematoma within the liver cyst, the increase in the nodule size and the patient's family history of cancer made it challenging to rule out liver cystadenocarcinoma. Although we considered a diagnosis through the puncture of the hepatic cyst, we were concerned about the potential risk of needle seeding in case the cyst was malignant. Thus, we decided to perform a surgical diagnostic resection. Preoperative computer simulations indicated that even after removing the hepatic cyst, the residual liver volume remained >800 mL. Therefore, an open‐right hepatectomy was planned.

Ultrasonography during surgery showed that the nodule had a consistently high echogenicity (Figure 2F), and a right hepatectomy was performed without any rupture along the cyst wall. The surgery lasted for 6 h and 29 min, with a blood loss of 745 mL. She started eating on postoperative day 4, and her liver function progressively improved. The postoperative course was uneventful, and the patient was discharged on day 16. Currently, the patient is undergoing outpatient follow‐up and has a recurrence‐free survival of 6 months.

**FIGURE 3:**
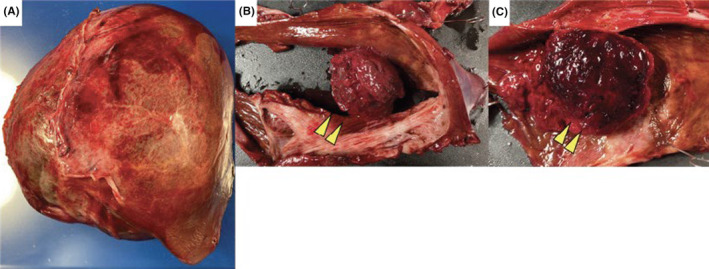
Macroscopic findings. (A) Resected liver cyst weighing approximately 900 g occupying the right liver lobe. (B) The cyst wall is entirely smooth, and the intracystic nodule originates from the cyst wall. (C) The intracystic nodule is dark reddish in appearance and filled with blood components.

**FIGURE 4:**
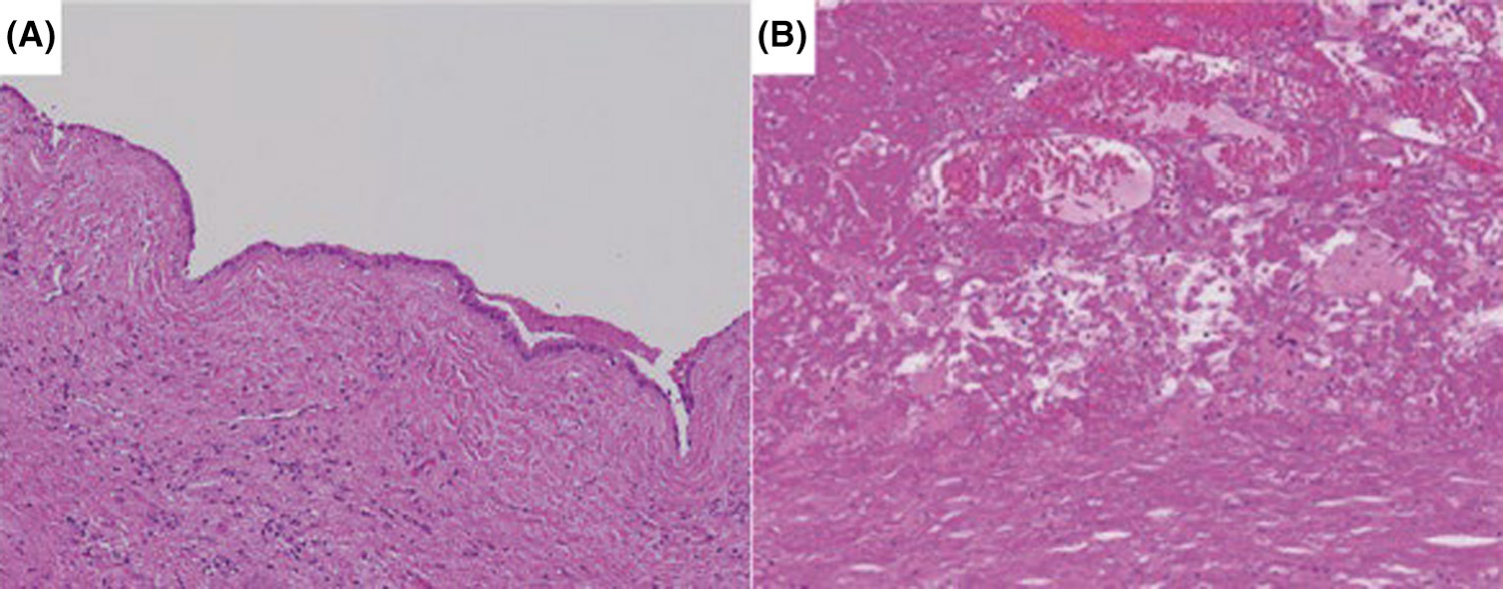
Pathological findings (×200). (A) No cell atypia is observed, and the layered structure of the liver cyst is preserved. (B) Tumor is mainly composed of erythrocytes and organizing fibrous components.

Overall, the 900‐g large liver cyst was soft (Figure 3A), with a white and smooth cyst wall and brownish cyst fluid. The cystic fluid had abnormally high CEA (517.3 ng/mL) and CA19‐9 (239,362 U/mL) levels; however, the nodules inside the cyst were uniformly dark red in color; therefore, malignancy was not actively suspected (Figure 3B,C). Histopathological findings showed no atypical cells in the cyst wall, with the layered structure preserved (Figure 4A), resulting in the diagnosis of a simple liver cyst with no evidence of malignancy or infectious diseases. The interior of the nodule primarily consisted of numerous erythrocytes and organizing fibrous components, which led to a final diagnosis of CEH within the liver cyst (Figure 4B).

## 3 DISCUSSION

In the present case, surgical resection was the treatment of choice for the liver cyst because of the difficulty in preoperatively determining its benign or malignant nature. This uncertainty was partly attributed to the infrequent appearance of nodules within liver cysts and limited number of reported cases.^4^, ^5^ Based on the pathological diagnosis, we considered this tumor to be a CEH originating within a liver cyst. A previous report defined CEH as a hematoma that gradually grows over a chronic course of more than 1 month.^3^ Although CEH is likely to occur in the limbs, there have been some reports of CEH in the liver that has been surgically resected.^6^, ^7^, ^8^ In this retrospective review, we examined several studies to determine the optimal approach for this case.

First, we considered differences in the frequency of occurrence. Liver cystadenocarcinoma accounts for only 0.12% of all primary liver malignancies,^9^ whereas intrahepatic hematoma reportedly occurs in approximately 10% of all hepatic cysts, making it relatively more common. However, chronic expanding hematomas, such as the one in this case, have a lower prevalence than liver hematomas. Furthermore, other liver tumors present with images similar to those in this case. Intraductal papillary neoplasms of the bile duct, mucinous cystic neoplasms of the liver, and *Echinococcus* parasitic infections are known to have similar imaging findings. These tumors are very difficult to distinguish through imaging. Given their prevalence among middle‐aged and older women, achieving an accurate diagnosis can be extremely difficult, even if their frequency varies. The diagnosis should be based on histopathological findings.

This also raises the question of how CEA and CA19‐9 levels in the cystic fluid affect diagnosis. In a previous study, CEA and CA19‐9 levels were measured in the cystic fluid of 40 patients with benign liver cysts and 32 patients with liver cystadenocarcinomas. In those with benign liver cysts, the CEA and CA19‐9 levels were 4.21 ± 2.91 (0.27–36.21) ng/mL and 21.37 ± 14.35 (0.60–85.92) U/mL, respectively, whereas in those with liver cystadenocarcinoma, the corresponding levels were 6.83 ± 2.43 (0.15–23.11) ng/mL and 21.37 ± 14.35 (0.60–85.92) U/mL, respectively, showing a higher tendency toward elevation in liver cystadenocarcinoma.^10^ However, there are no reports on the measurement of CEA or CA19‐9 levels in the cystic fluid of hematomas within liver cysts. In this case, the high CEA and CA19‐9 levels in the cystic fluid may have been caused by bleeding or hematoma formation, as the final pathological examination confirmed the nodule to be benign. Furthermore, other studies have reported that 35% of cytological diagnoses of cystic fluid puncture in cystadenocarcinoma of the liver are false negatives.^11^ There is also a concern that performing a puncture to differentiate benign from malignant lesions may increase the risk of seeding in malignant cases. Other side effects such as massive bleeding or bile leakage into the peritoneal space may be a concern. Given these considerations, the preoperative measurement of CEA and CA19‐9 levels in liver cystic fluid is currently of limited significance and high risk.

Thus, considering the limitations of imaging and potential risk of puncture, *en bloc* surgical resection remains the first choice of treatment and diagnosis for nodules within liver cysts. However, if the disease is benign, as in the present case, there is little need for surgical resection, which would be an overinvasive hepatic resection. The timing of treatment and the balance between its advantages and disadvantages must be carefully considered. Liver cystadenocarcinoma has been reported to have a better prognosis than primary liver cancers, such as hepatocellular carcinoma and intrahepatic cholangiocarcinoma, with a 5‐year survival rate of >70% after complete resection.^1^ Although no clear criteria have been established, it is important to determine whether surgical resection should be performed by examining the patient's general condition and the benefits and risks of the treatment. We hope that our experience will assist in the optimal treatment of intrahepatic cystic nodules.

## AUTHOR CONTRIBUTIONS

**Kodai Abe:** Conceptualization; resources; writing – original draft. **Shunsuke Tomita:** Data curation; validation. **Shohei Suzuki:** Formal analysis; visualization. **Yu Kigasawa:** Formal analysis; investigation; visualization. **Hironobu Kashiwagi:** Methodology; resources; visualization. **Michihito Nishioka:** Methodology; validation; writing – review and editing. **Yasuo Kabeshima:** Project administration; supervision.

## FUNDING INFORMATION

Not applicable.

## CONFLICT OF INTEREST STATEMENT

The authors declare that they have no conflict of interest.

## ETHICS STATEMENT

All procedures followed were in accordance with the ethical standards of the responsible committee on human experimentation and with the Helsinki Declaration of 1975, as revised in 2008.

## CONSENT

Written informed consent was obtained from the patient to publish this report in accordance with the journal's patient consent policy.

## Data Availability

All data generated or analyzed during this study are included in this published article.
